# Olfactory sensitivity for mold-associated odorants in CD-1 mice and spider monkeys

**DOI:** 10.1007/s00359-018-1285-x

**Published:** 2018-09-10

**Authors:** Luis Peixoto, Laura Teresa Hernandez Salazar, Matthias Laska

**Affiliations:** 1 IFM Biology, Linköping University, 581 83 Linköping, Sweden; 2 Instituto de Neuro-Etologia, Universidad Veracruzana, C.P. 91000 Xalapa, Veracruz Mexico

**Keywords:** Olfactory detection thresholds, Mold-associated odorants, CD-1 mice, Spider monkeys, Olfactory sensitivity

## Abstract

Using operant conditioning procedures, we assessed the olfactory sensitivity of six CD-1 mice and three spider monkeys for mold-associated odorants. We found that with all eight stimuli, the mice detected concentrations as low as 0.1 ppm (parts per million), and with two of them individual animals even detected concentrations as low as 1 ppt (parts per trillion). The spider monkeys detected concentrations as low as 4 ppm with all eight stimuli, and with four of them individual animals even detected concentrations as low as 4 ppb (parts per billion). Between-species comparisons showed that with all eight odorants, the mice displayed significantly lower threshold values, that is, a higher sensitivity than the spider monkeys, but not than human subjects tested in previous studies. Analysis of odor structure–activity relationships showed that in both species, the type of oxygen-containing functional group and the presence versus absence of a double bond as well as the length of the carbon backbone of the odor stimuli had a systematic effect on detectability. We conclude that both mice and spider monkeys are clearly able to detect the presence of molds and thus to assess the palatability of potential food using the volatiles produced by molds during putrefaction.

## Introduction

Food selection is the process by which animals try to optimize their energy yield, to meet their nutrient requirements, and to avoid ingestion of potentially harmful substances (Stephens and Krebs 1986). The vast majority of terrestrial mammal species rely on, or at least include, their sense of smell for food selection (Stoddart 1980; Hughes 1990). This should not be surprising given that fruits, for example, systematically change their odor in the course of maturation and thus provide an honest chemical signal of their nutritional value (Goff and Klee 2006; Nevo and Valenta 2018). Similarly, the odor of animal prey changes systematically over time and thus allows predators or scavengers to assess its palatability (Vass et al. 2004; Forbes and Perrault 2014).

One of the most challenging tasks in the food selection process is for an animal to detect whether a potential food item is spoiled or rotten due to putrefaction, that is, the microbial decomposition of organic matter (Janzen 1977) and accordingly, to decide for or against consumption. In addition to bacteria, fungi such as molds are the major contributors to the degradation of proteins and thus to food spoilage (Pitt and Hocking 2009). Molds comprise a large and taxonomically diverse group of fungal species that grow in the form of filaments or hyphae. The ingestion of mold-infested food can cause severe health effects or even death due to highly toxic secondary metabolites such as aflatoxins or ergot alkaloids produced by molds (Murphy et al. 2006). Chemo-analytical studies have identified a number of volatiles that are a product of the microbial degradation of food and which humans perceive and describe as “moldy” (Kaminski et al. 1974; Chambers et al. 1998). The quantitatively predominant mold-associated odorants found in the headspace above fungal species such as *Aspergillus, Penicillium*, and *Fusarium* include, but are not restricted to, branched and/or unsaturated aliphatic ketones and alcohols (Börjesson et al. 1992; Schnürer et al. 1999; Li et al. 2016).

It seems reasonable to assume that mammals feeding on plant material that is possibly infested by molds should be sufficiently sensitive to detect such mold-associated odorants to avoid ingestion. This should be particularly important for mammal species lacking or having insufficient gastrointestinal detoxification mechanisms for aflatoxins and other harmful metabolites of molds (Wong and Hsieh 1980). It was, therefore, the aim of the present study to determine olfactory detection thresholds in a granivorous rodent, the house mouse (*Mus musculus*), and in a frugivorous primate, the spider monkey (*Ateles geoffroyi*), for an array of mold-associated odorants. It is well established that both species strongly rely on their sense of smell in the context of food selection (Laska et al. 2007; Morris et al. 2012; Nevo et al. 2015; Pablo-Rodriguez et al. 2015) and that both grains and fruits are prime targets of mold infestation (Jelen and Wasowicz 1998; Barkai-Golan and Paster 2008). A between-species comparison of olfactory detection thresholds allowed us to evaluate whether neuroanatomical properties such as the size of olfactory brain structures or genetic properties such as the number of functional olfactory receptor genes correlate with olfactory sensitivity. To this end, we also compared the data obtained in the present study with data obtained with human subjects tested on the same set of odorants in previous studies. The fact that several of the odorants under investigation are structurally related and only differ from each other, e.g., in the type of oxygen-containing functional group, the presence versus absence of a double bond, or the length of the carbon backbone allowed us to additionally assess the impact of these molecular structural features on olfactory detectability.

Specifically, we tested the following predictions: (1) neither neuroanatomical nor genetic properties of a species are systematically linked with olfactory sensitivity; (2) molecular structural features of the odorants under investigation have a systematic effect on olfactory detectability; and (3) olfactory sensitivity is positively linked with the behavioral relevance of the odor stimuli.

## Materials and methods

### Animals

Testing was carried out using six male CD-1 mice (*Mus musculus*) and two female and one male spider monkeys (*Ateles geoffroyi*). The rationale for choosing this outbred strain of mice was to use animals with a genetic background that is diverse and more similar to that of wild mice than that of any inbred strain (Crusio et al. 2013). Care was taken to use animals that were not litter mates to minimize the probability of genetic similarity between individuals. The mice were 150–170 days at the beginning of the study.

The rationale for choosing spider monkeys was that data on olfactory detection thresholds for homologous series of aliphatic aldehydes (Laska et al. 2006b, c), carboxylic acids (Laska et al. 2004; Güven and Laska 2012), and ketones (Eliasson et al. 2015; Laska 2014), as well as for structurally related aromatic aldehydes (Larsson and Laska 2011; Kjeldmand et al. 2011), alkylpyrazines (Laska et al. 2009), monoterpenes (Joshi et al. 2006), “green” odors (Løtvedt et al. 2012), amino acids (Wallén et al. 2012), and sulfur-containing predator odorants (Sarrafchi et al. 2013) were obtained in earlier studies with both species, allowing for direct between-species comparisons of olfactory sensitivity. Furthermore, information about neuroanatomical and genetic properties of both species is at hand (Stephan et al. 1988; Gilad et al. 2004; Hughes et al. 2018). The spider monkeys were 4, 9, and 11 years of age, respectively, at the beginning of the study. Maintenance of both species has been described in detail elsewhere (mice: Laska et al. 2006b, 2003a, b; spider monkeys:).

### Odorants

**Fig. 1:**
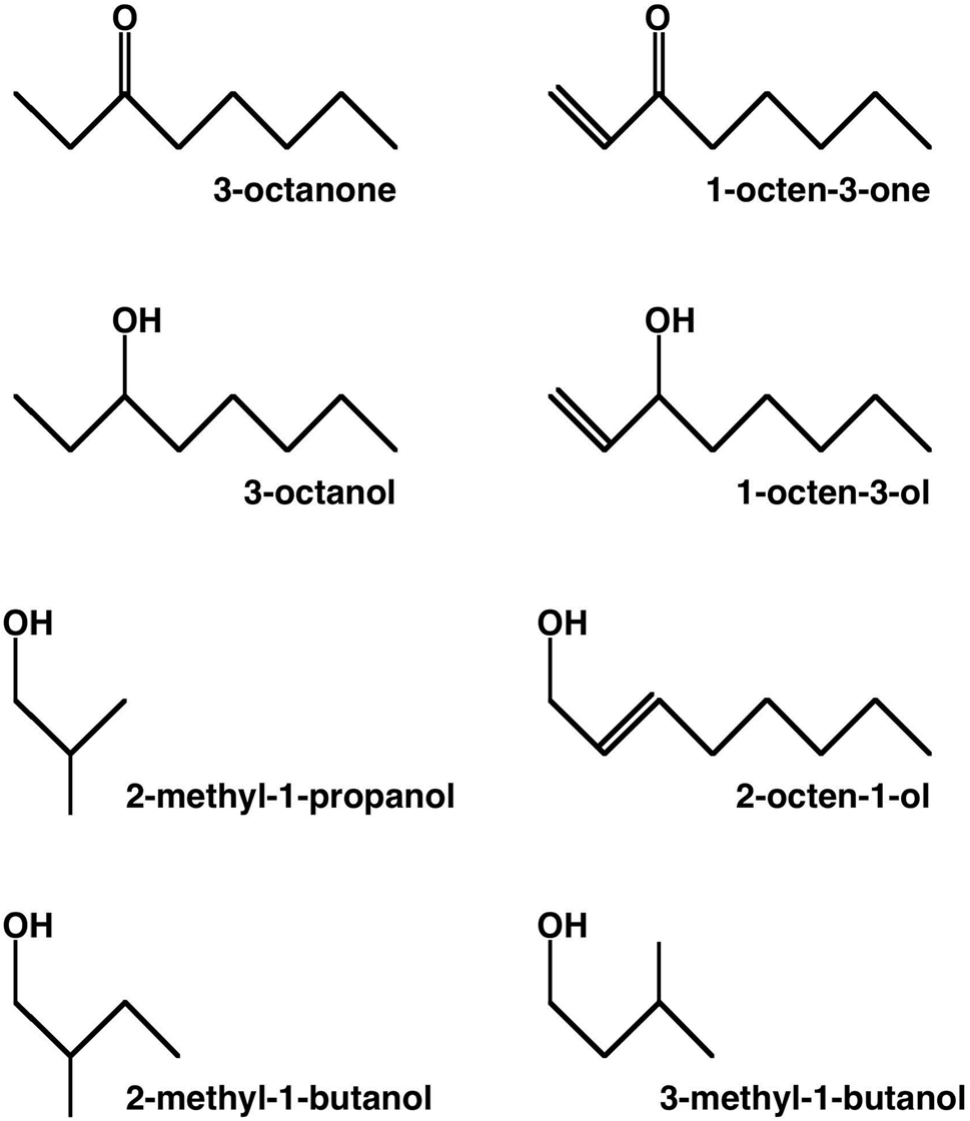
Chemical structure of the eight mold-associated odorants used

A set of eight odorants was used: 1-octen-3-ol (CAS# 3391-86-4), 1-octen-3-one (CAS# 4312-99-6), 3-octanol (CAS# 589-98-0), 3-octanone (CAS# 106-68-3), trans-2-octen-1-ol (CAS# 18409-17-1), 2-methyl-1-propanol (CAS# 78-83-1), 2-methyl-1-butanol (CAS# 137-32-6), and 3-methyl-1-butanol (CAS# 123-51-3). The rationale for choosing these substances was to assess the olfactory sensitivity of the mice and spider monkeys for odorants known to be quantitatively predominant components of the odor produced by molds (Börjesson et al. 1992; Schnürer et al. 1999). All substances were obtained from Sigma-Aldrich (St. Louis, MO) and had a nominal purity of at least 99.5%. They were diluted using near-odorless diethyl phthalate (CAS# 84-66-2) as the solvent. Gas-phase concentrations of the headspace above the diluted odorants were calculated using published vapor pressure data (Dykyi et al. 2001) and corresponding formulae (Weast 1987), and were empirically verified using a miniPID Gas Sensor (Fast Response Miniature Photo-ionization Detector, model 200B, Aurora Scientific, Aurora, ON, Canada). Figure 1 shows the molecular structure of the odorants.

### Behavioral test

Olfactory sensitivity of the mice was assessed using an automated liquid-dilution olfactometer (Knosys, Tampa, FL) and an instrumental conditioning go/no-go procedure which has been described in detail elsewhere (Bodyak and Slotnick 1999). Briefly, animals were trained to insert their snout into the odor sampling port of a test chamber. This triggered a 2 s presentation of either an odorant used as the rewarded stimulus (S+) or a blank (headspace of the solvent) used as the unrewarded stimulus (S–). Licking at a steel tube providing 2.5 µl of water reinforcement in response to presentation of the S + served as the operant response. Not licking in response to presentation of the S- was not rewarded. Incorrect responses were not punished. Forty such trials (20 S + and 20 S– trials in pseudorandomized order) using the same concentration of the S + were conducted per animal and condition. A total of 120 trials, subdivided into six blocks of 20 trials each, were performed per animal and day. To ensure reliable cooperation, the animals were kept on a water deprivation schedule of 1.5 ml of water per day. Part of this amount of water was consumed during the test session, and the rest was fed to the animal immediately after the end of the test session.

Olfactory detection thresholds were determined by testing the animals’ ability to discriminate between increasing dilutions of the odorant used as S+, and the solvent alone used as S–. Starting with a gas-phase concentration of 1 ppm (parts per million), each stimulus was successively presented in tenfold dilution steps until an animal failed to significantly discriminate the odorant from the solvent. Subsequently, an intermediate concentration (0.5 log units between the lowest concentration that was detected above chance and the first concentration that was not) was tested to determine the threshold value more exactly.

Olfactory sensitivity of the spider monkeys was assessed using a food-rewarded instrumental conditioning procedure which has been described in detail elsewhere (Laska et al. 2003a). Briefly, the animals were trained to sniff at two simultaneously presented boxes equipped with absorbent paper strips that were impregnated with 20 µl of an odorant or the near-odorless solvent signalling either that they contained a food reward (S+) or that they did not (S–). Opening of one of the boxes served as the operant response. 30 such trials (15 S + and 15 S– trials in pseudorandomized order) using the same concentration of a given S + were conducted per animal and condition.

Olfactory detection thresholds were determined by testing the animals’ ability to discriminate between increasing dilutions of an odorant used as S+, and the near-odorless solvent alone used as S–. Starting with a 100-fold liquid dilution, each stimulus was successively presented in 10-fold dilution steps until an animal failed to significantly discriminate the odorant from the solvent. Subsequently, an intermediate concentration (0.5 log units between the lowest concentration that was detected above chance and the first concentration that was not) was tested to determine the threshold value more exactly.

### Data analysis

For each individual animal, the percentage of correct choices from 40 (mice) and 30 (spider monkeys) trials per dilution step was calculated. With the mice, correct choices consisted both of licking in response to presentation of the S + and not licking in response to the S–, and errors consisted of animals showing the reverse pattern of operant responses, that is: not licking in response to the S + and licking in response to the S–. With the spider monkeys, correct choices consisted both of animals opening a box equipped with the S + and failing to open a box equipped with the S−. Conversely, errors consisted of animals opening a box equipped with the S− or failing to open a box equipped with the S+. Significance levels were determined by calculating binomial *z*-scores corrected for continuity from the number of correct and false responses for each individual and condition. All tests were two-tailed and the alpha level was set at 0.01. Possible differences in sensitivity between the two species were assessed using the Mann–Whitney *U* test for independent samples. Possible differences in sensitivity between odorants were assessed using the Wilcoxon signed-rank test for related samples. Bonferroni corrections were used to counteract the problem of multiple comparisons wherever appropriate.

## Results

### CD-1 mice

**Fig. 2:**
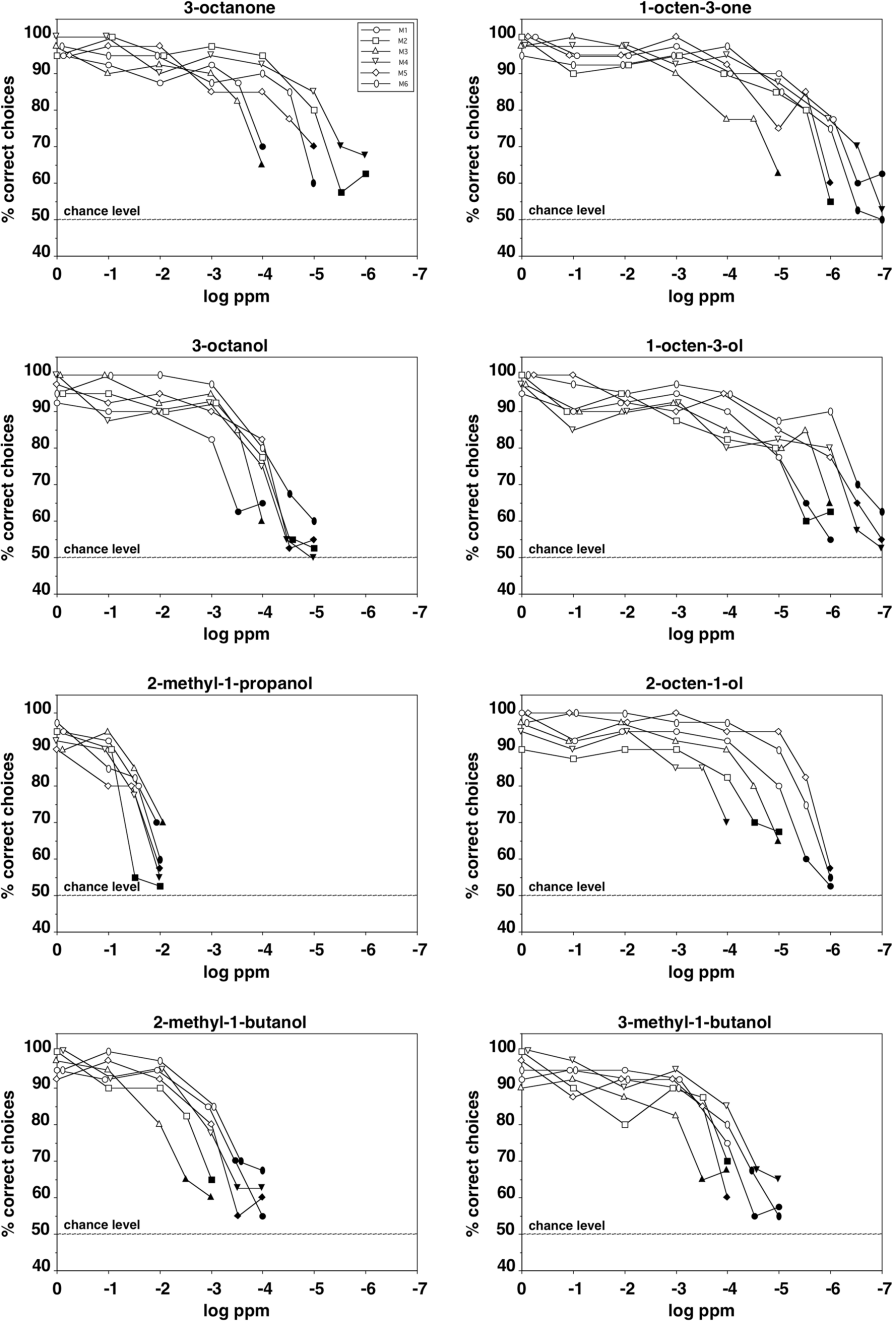
Performance of the six CD-1 mice in discriminating between various concentrations of a mold-associated odorant and the solvent. Each data point represents the percentage of correct choices from a total of 40 decisions per individual animal. The six different symbols represent data from each of the six individual animals tested per odorant. Filled symbols indicate concentrations that were not discriminated significantly above chance level (binomial test, *p* > 0.01)

Figure 2 shows the performance of the mice in discriminating between various concentrations of a given odorant and the solvent. With all eight odorants, all six animals significantly distinguished concentrations as low as 0.1 ppm (parts per million) from the solvent (binomial test, *p* < 0.01) and with two of them (1-octen-3-one and 1-octen-3-ol) individual animals even detected concentrations as low as 1 ppt (parts per trillion).

The individual mice generally demonstrated similar detection threshold values with a given odorant and with four of the eight odorants (1-octen-3-ol, 3-octanol, 2-methyl-1-butanol, and 3-methyl-1-butanol) they differed only by a dilution factor of 10 between the most- and the least-sensitive animal. With one odorant (2-methyl-1-propanol), the range of threshold values was even only a dilution factor of 3. The largest difference in sensitivity for a given odorant between individuals was a dilution factor of 100 and was found with 2-octen-1-ol.

Table 1 summarizes the threshold values of the mice for the eight odorants and shows various measures of corresponding gas-phase concentrations allowing readers to easily compare the data obtained in the present study tho those reported by other authors using one of these convertible measures.

**Table 1:** Olfactory detection threshold values for mold-associated odorants in CD-1 mice, expressed in various measures of gas-phase concentrations

	*n*	Molec./cm^3^ air	ppm	Log ppm	Mol/l	Log Mol/l
1-octen-3-one	1	7.5 × 10^8^	0.00003	− 4.52	1.3 × 10–12	− 11.87
	2	7.5 × 10^7^	0.000003	− 5.52	1.3 × 10^–13^	− 12.87
	3	2.5 × 10^7^	0.000001	− 6.00	4.5 × 10^–14^	− 13.35
1-octen-3-ol	2	2.5 × 10^8^	0.00001	− 5.00	4.5 × 10^–13^	− 12.35
	1	7.5 × 10^7^	0.000003	− 5.52	1.3 × 10^–13^	− 12.87
	3	2.5 × 10^7^	0.000001	− 6.00	4.5 × 10^–14^	− 13.35
3-octanone	2	7.5 × 10^9^	0.0003	− 3.52	1.3 × 10^–11^	− 10.87
	2	7.5 × 10^8^	0.00003	− 4.52	1.3 × 10^–12^	− 11.87
	2	2.5 × 10^8^	0.00001	− 5.00	4.5 × 10^–13^	− 12.35
3-octanol	1	2.5 × 10^10^	0.001	− 3.00	4.5 × 10^–11^	− 10.35
	1	7.5 × 10^9^	0.0003	− 3.52	1.3 × 10^–11^	− 10.87
	4	2.5 × 10^9^	0.0001	− 4.00	4.5 × 10^–12^	− 11.35
2-octen-1-ol	1	7.5 × 10^9^	0.0003	− 3.52	1.3 × 10^–11^	− 10.87
	1	2.5 × 10^9^	0.0001	− 4.00	4.5 × 10^–12^	− 11.35
	1	7.5 × 10^8^	0.00003	− 4.52	1.3 × 10^–12^	− 11.87
	1	2.5 × 10^8^	0.00001	− 5.00	4.5 × 10^–13^	− 12.35
	2	7.5 × 10^7^	0.000003	− 5.52	1.3 × 10^–13^	− 12.87
2-methyl-1-propanol	1	2.5 × 10^12^	0.1	− 1.00	4.5 × 10^−9^	− 8.35
	5	7.5 × 10^11^	0.03	− 1.52	1.3 × 10^−9^	− 8.87
2-methyl-1-butanol	1	2.5 × 10^11^	0.01	− 2.00	4.5 × 10^–10^	− 9.35
	1	7.5 × 1010	0.003	− 2.52	1.3 × 10^–10^	− 9.87
	4	2.5 × 10^10^	0.001	− 3.00	4.5 × 10^–11^	− 10.35
3-methyl-1-butanol	1	2.5 × 10^10^	0.001	− 3.00	4.5 × 10^–11^	− 10.35
	2	7.5 × 10^9^	0.0003	− 3.52	1.3 × 10^–11^	− 10.87
	3	2.5 × 10^9^	0.0001	− 4.00	4.5 × 10^–12^	− 11.35

*n* number of animals, *ppm* parts per million

### Spider monkeys

**Fig. 3:**
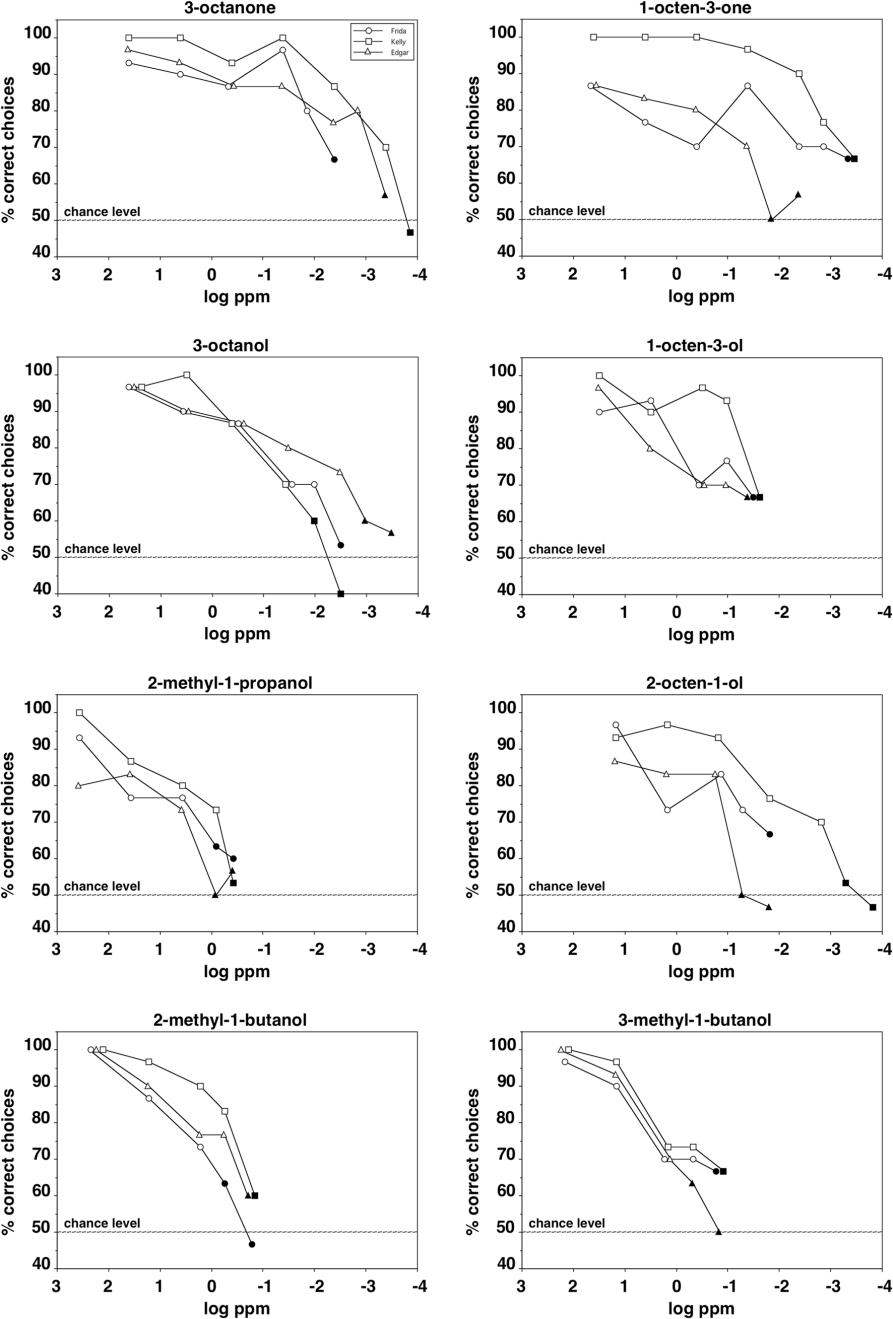
Performance of the three spider monkeys in discriminating between various concentrations of a mold-associated odorant and the solvent. Each data point represents the percentage of correct choices from a total of 30 decisions per individual animal. The three different symbols represent data from each of the three individual animals tested per odorant. Filled symbols indicate concentrations that were not discriminated significantly above chance level (binomial test, *p* > 0.01)

Figure 3 shows the performance of the spider monkeys in discriminating between various concentrations of a given odorant and the solvent. With all eight odorants, all three animals distinguished concentrations as low as 4 ppm (parts per million) from the solvent (binomial test, *p* < 0.01) and with four of them (1-octen-3-one, 3-octanone, 3-octanol, and 2-octen-1-ol) individual animals even detected concentrations as low as 4 ppb (parts per billion).

The individual spider monkeys generally demonstrated similar detection threshold values with a given odorant and with four of the eight odorants they differed only by a dilution factor of 3 (2-methyl-1-propanol, 2-methyl-1-butanol, 3-methyl-1-butanol) or 10 (3-octanol) between the most- and the least-sensitive animal. With one odorant (1-octen-3-ol), all three animals even scored the same threshold value. The largest difference in sensitivity for a given odorant between individuals was a dilution factor of 100 and was found with 2-octen-1-ol. Table 2 summarizes the threshold values of the mice for the eight odorants and shows various measures of corresponding gas-phase concentrations.

**Table 2:** Olfactory detection threshold values for mold-associated odorants in spider monkeys, expressed in various measures of gas-phase concentrations

	*n*	Molec./cm^3^ air	ppm	Log ppm.	Mol/l	Log Mol/l
1-octen-3-one	1	1.1 × 10^12^	0.041	− 1.39	1.8 × 10^−9^	− 8.74
	2	3.7 × 10^10^	0.0014	− 2.86	6.1 × 10^–11^	− 10.21
1-octen-3-ol	3	2.8 × 10^12^	0.10	− 0.98	4.6 × 10^−9^	− 8.33
3-octanone	1	3.7 × 10^11^	0.014	− 1.86	6.1 × 10^–10^	− 9.21
	1	3.7 × 10^10^	0.0014	− 2.86	6.1 × 10^–11^	− 10.21
	1	1.1 × 10^10^	0.00041	− 3.39	1.8 × 10^–11^	− 10.74
3-octanol	1	8.5 × 10^11^	0.031	− 1.50	1.4 × 10^−9^	− 8.85
	1	2.8 × 10^11^	0.010	− 1.98	4.6 × 10^–10^	− 9.33
	1	8.5 × 10^10^	0.0031	− 2.50	1.4 × 10^–10^	− 9.85
2-octen-1-ol	1	4.1 × 10^12^	0.15	− 0.82	6.8 × 10^−9^	− 8.17
	1	1.4 × 10^12^	0.052	− 1.29	2.3 × 10^−9^	− 8.63
	1	4.1 × 10^10^	0.0015	− 2.82	6.8 × 10^–11^	− 10.17
2-methyl-1-propanol	2	1.0 × 10^14^	3.70	0.57	1.7 × 10^−7^	− 6.78
	1	3.3 × 10^13^	1.22	0.09	5.5 × 10^−8^	− 7.26
2-methyl-1-butanol	1	4.5 × 10^13^	1.67	0.22	7.5 × 10^−8^	− 7.13
	2	1.5 × 10^13^	0.56	− 0.26	2.5 × 10^−8^	− 7.60
3-methyl-1-butanol	1	3.9 × 10^13^	1.44	0.16	6.5 × 10^−8^	− 7.19
	2	1.3 × 10^13^	0.48	− 0.32	2.2 × 10^−8^	− 7.67

*n* number of animals, *ppm* parts per million

### Comparison between species

**Fig. 4:**
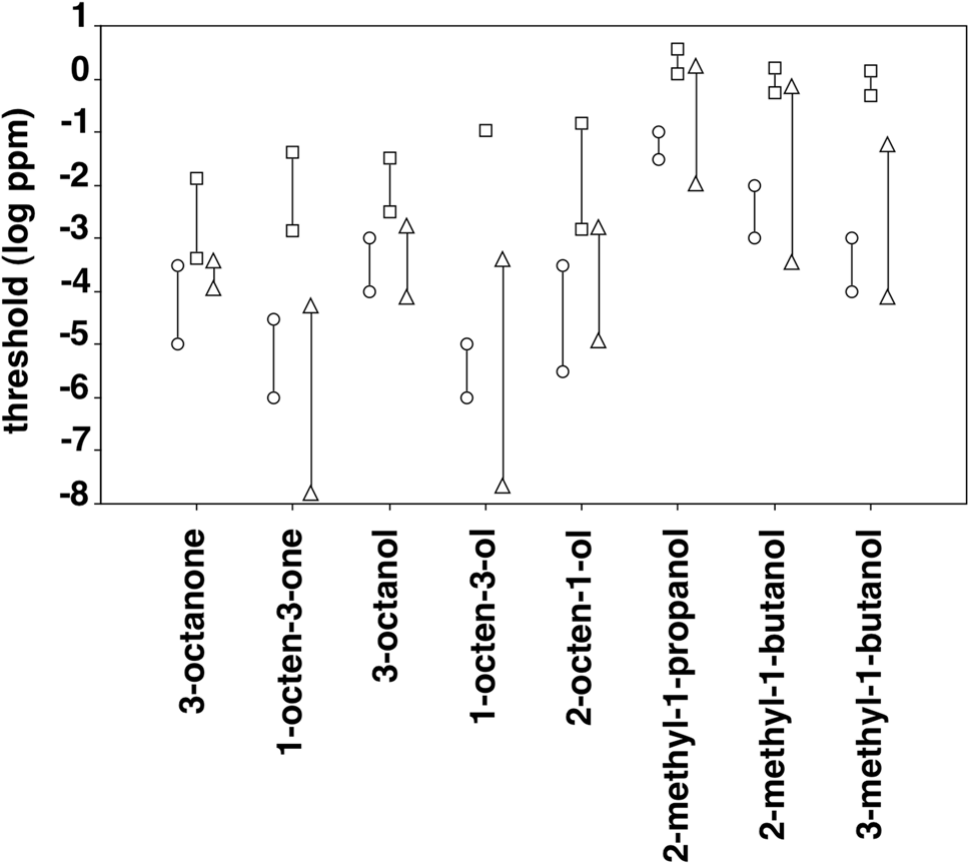
Comparison of the olfactory detection thresholds of the CD-1 mice and the spider monkeys for the eight mold-associated odorants and those of human subjects. Data points of the mice (circles) and the spider monkeys (squares) represent the highest and the lowest threshold values of individual animals. Data points of the human subjects (triangles) represent the highest and the lowest mean threshold values reported in the literature (van Gemert 2011)

Figure 4 compares the ranges of olfactory detection threshold values of the mice and the spider monkeys obtained in the present study and corresponding data of human subjects obtained in previous studies for the eight mold-associated odorants tested. With all eight odorants, all six mice displayed significantly lower threshold values, that is, a higher sensitivity than the three spider monkeys (Mann–Whitney *U* test, *p* < 0.05 with all eight odorants). Accordingly, the ranges of threshold values with a given odorant did not overlap between *Mus musculus* and *Ateles geoffroyi*. A comparison between the olfactory detection threshold values of the mice and those of human subjects tested in previous studies (van Gemert 2011) shows that the ranges of threshold values overlap between the two species with all eight odorants. With six of the eight odorants (1-octen-3-one, 3-octanol, 1-octen-3-ol, 2-methyl-1-propanol, 2-methyl-1-butanol, and 3-methyl-1-butanol), the lowest mean human threshold value was even lower than the lowest individual mouse threshold value. (Please note that human studies usually do not report threshold values of individual subjects, but only mean threshold values across groups of subjects. Thus, statistical comparisons between the olfactory sensitivity of humans and mice are not possible.) A corresponding comparison between the olfactory detection threshold values of the spider monkeys and of human subjects shows that the ranges of threshold values overlap between the two species with four of the odorants (3-octanone, 2-octen-1-ol, 2-methyl-1-propanol, and 2-methyl-1-butanol). With the remaining four odorants (1-octen-3-one, 3-octanol, 1-octen-3-ol, and 3-methyl-1-butanol), *Homo sapiens* displays lower threshold values than *Ateles geoffroyi*.

### Odorant structure–activity relationships

A comparison of the olfactory detection threshold values between odorants which only differed from each other in their functional oxygen-containing group (3-octanone versus 3-octanol, and 1-octen-3-one versus 1-octen-3-ol) showed that both the mice and the spider monkeys were significantly more sensitive for the ketones than for the corresponding alcohols (Wilcoxon, *p* < 0.05 with both species). Similarly, the presence versus the absence of a double bond (3-octanone versus 1-octen-3-one, and 3-octanol versus 1-octen-3-ol) had a systematic effect on olfactory detection thresholds in both species: the mice were significantly more sensitive for odorants with a double bond compared to odorants without a double bond, whereas the spider monkeys displayed the opposite pattern of sensitivity (Wilcoxon, *p* < 0.05 with both species). The relative position of the double bond and the functional alcohol group towards each other (1-octen-3-ol versus 2-octen-1-ol) and the position of the methyl group (2-methyl-1-butanol versus 3-methyl-1-butanol) had a systematic effect on sensitivity in the mice (Wilcoxon, *p* < 0.05 with both odor pairs), but not in the spider monkeys (Wilcoxon, *p* > 0.05 with both odor pairs). Finally, the length of the carbon backbone systematically affected olfactory detection thresholds, with both mice and spider monkeys being significantly more sensitive for alcohols with a C_8_ backbone (1-octen-3-ol, 3-octanol, and 2-octen-1-ol) compared to alcohols with a C_4_ or C_5_ backbone (2-methyl-1-propanol, 2-methyl-1-butanol, and 3-methyl-1-butanol) (Wilcoxon, *p* < 0.05 with both species).

## Discussion

The results of the present study show that mice and spider monkeys have a well-developed olfactory sensitivity for mold-associated odorants. Furthermore, they demonstrate that mice are significantly more sensitive for this group of volatiles than spider monkeys and that molecular structural features of the odorants had a systematic effect on their detectability in both species.

### Olfactory sensitivity

The interindividual variability of the detection threshold values for a given odorant and a given species in the present study was low and considerably smaller than the range reported in studies on human olfactory sensitivity (Doty and Laing 2015). With the majority of odorants tested, the largest difference between the most- and the least-sensitive animal of a given species was a dilution factor of 10 or lower (see Figs. 2, 3). Therefore, the results can be considered as robust, even though only six mice and three spider monkeys, respectively, were tested. Furthermore, the animals’ performance with the lowest concentrations presented dropped to chance level, suggesting that the statistically significant discrimination between higher concentrations of an odorant and the solvent was indeed based on chemosensory perception and not on other cues.

### Between-species comparisons

A between-species comparison of the threshold values obtained with the mice and the spider monkeys in the present study shows that the former clearly outperformed the latter with all eight mold-associated odorants. Interestingly, however, the mice were not more sensitive compared to human subjects, the only other species for which olfactory detection thresholds with the mold-associated odorants tested here have been reported so far (see Fig. 4). This raises the question as to possible mechanisms underlying the observed differences and similarities in olfactory sensitivity between species.

Several studies have tried to link between-species differences in olfactory sensitivity to neuroanatomical properties such as the relative or the absolute size of olfactory brain structures (Nummela et al. 2013). The present findings only partially support the existence of such a link: our finding that the mice were clearly more sensitive for the mold-associated odorants than the spider monkeys fits to the fact that the relative size of the olfactory bulbs in *Mus musculus* (2.0% of total brain volume, Kovacevic et al. 2005) is clearly larger than that of *Ateles geoffroyi* (0.09% of total brain volume, Stephan et al. 1988). However, the finding that humans were at least as sensitive for these odor stimuli as the mice does not fit as the relative size of the olfactory bulbs in *Homo sapiens* (0.01%, Stephan et al. 1988) is the smallest of the three species. Similarly, a comparison of the absolute size of the olfactory bulbs in the three species does not support the notion of a positive correlation between this neuroanatomical property and olfactory sensitivity: mice have markedly smaller olfactory bulbs in terms of absolute size (8.3 mm^3^, Pomeroy et al. 1990) compared to both spider monkeys (90.4 mm^3^, Stephan et al. 1988) and human subjects (114 mm^3^, Stephan et al. 1988). Previous studies also yielded inconclusive findings in this respect, with some of them supporting the notion of a positive correlation between the relative or the absolute size of the olfactory bulbs and a species’ olfactory sensitivity and some of them failing to do so (Hernandez Salazar et al. 2003; Joshi et al. 2006; Kjeldmand et al. 2011; Løtvedt et al. 2012). Future studies should, therefore, explore the possibility that the degree of neural connectivity rather than the absolute or relative numbers of neurons involved in olfactory processing may be relevant for the sensitivity of olfactory systems (Keverne 2004; Shepherd 2005).

Other studies proposed that between-species differences in olfactory sensitivity might be linked to genetic properties such as the number of functional or the proportion of non-functional olfactory receptor genes (Rouquier and Giorgi 2007). Here, too, the findings of the present study only partially support this notion as mice have a markedly higher number of functional genes coding for olfactory receptors (≈ 1230, Hughes et al. 2018) compared to both spider monkeys (≈ 900, Gilad et al. 2004) and human subjects (≈ 390, Hughes et al. 2018), but nevertheless, the mice were not more sensitive than the human subjects for the odorants tested here. Similarly, although mice have been reported to have a markedly lower proportion of non-functional olfactory receptor genes (11.2%, Hughes et al. 2018) compared to spider monkeys (18.4%, Gilad et al. 2004) and human subjects (54.4%, Hughes et al. 2018), their olfactory detection thresholds for the mold-associated odorants did not systematically differ from those of the human subjects. This suggests that it may not necessarily be the absolute or the relative size, but perhaps, the composition of the repertoire of functional olfactory receptor genes that may determine a species’ olfactory sensitivity for a given odorant (Nei et al. 2008; Hughes et al. 2018). In summary, these findings are in line with our first prediction that neither neuroanatomical nor genetic properties of a species are systematically linked with olfactory sensitivity.

Whether between-species differences in the ability to detoxify harmful mold-associated metabolites such as aflatoxins might underlie the observed differences between mice, spider monkeys, and humans in their olfactory sensitivity for mold-associated odorants is difficult to decide. The LD50 of mice for Aflatoxin B1, for example, is 9.0 mg/kg of body weight, and thus, 25-times higher than the lethal dose of 0.36 mg/kg reported for this substance in humans (Dhanasekaran et al. 2011). No corresponding data for spider monkeys are available. Future studies should, therefore, explore possible links between olfactory sensitivity for odorants which are either toxic themselves or indicative of toxic food and the effectiveness of an organism’s detoxification mechanisms.

Finally, although we cannot completely exclude the possibility that differences between methods may have affected the animals’ performance, and thus, the comparability of our results, it should be considered that most of these differences are necessary adaptations to meet the physiological, anatomical, and behavioral needs and limitations of our study species to successfully cooperate in a behavioral test of sensory capabilities (Hastings 2003). Both the mice and the spider monkeys were tested using instrumental conditioning procedures which are commonly regarded as the gold standard in animal psychophysics (Pearce 2008) and which virtually exclude the possibility that differences in motivation may underlie the observed between-species differences in olfactory sensitivity. Similarly, we consider it as unlikely that the different modes of odor stimulus presentation used in the present study, an olfactometer with the mice and odorized paper strips with the spider monkeys, systematically affected our findings. If that was the case, then it would be difficult to explain that spider monkeys have been reported to be more sensitive than mice, using exactly the same methods as in the present study, with at least 11 odorants that have been tested with both species (Laska and Hernandez Salazar 2015; Laska 2017). Furthermore, we used a photo-ionization detector with both modes of odor presentation and verified that the gas-phase concentrations in the headspace above the odorants that we calculated using published vapor pressure data (Dykyi et al. 2001) and classical gas law formulae (Weast 1987) corresponded well with the empirically obtained data.

All the factors mentioned above that may, or may not, affect the comparability of olfactory detection thresholds across species also apply to the comparisons between the olfactory sensitivity of our two study species and human subjects. The human data shown in Fig. 4, for example, were obtained using different modes of stimulus presentation which ranged from squeeze bottles to olfactometers (van Gemert 2011). Here, too, the authors tried their best to provide accurate gas-phase concentrations. Keeping all these caveats in mind, we still feel that between-species comparisons provide a valuable means to assess the neuroanatomical or genetic mechanisms that have been proposed to underlie between-species differences in olfactory sensitivity and are indispensable to elucidate the selective pressures acting on the olfactory systems of different species.

### Odor structure–activity relationships

Both mice and spider monkeys displayed lower detection threshold values with the ketones tested here compared to the corresponding alcohols. This is in line with several previous studies which reported that the type of oxygen-containing functional group had a systematic effect on olfactory detectability (mice: Güven and Laska 2012; Laska 2014; spider monkeys:; Hernandez Salazar et al. 2003; Laska et al. 2006c). However, it is interesting to note that spider monkeys have also been reported to be more sensitive to aliphatic 1-alcohols than to aliphatic 2-ketones (Eliasson et al. 2015). This finding, however, does not necessarily contradict the current results, but rather shows that molecular structural features such as the position of the functional group—which differs between the odorants used in the two studies—may also affect an odorant’s detectability.

Furthermore, we found that the presence versus the absence of a double bond also had a systematic effect on olfactory detection thresholds in both species tested. Previous studies failed to find such an effect in both mice and spider monkeys when tested with aliphatic C_6_-alcohols (Løtvedt et al. 2012) or acyclic monoterpene alcohols having or lacking a double bond (Laska et al. 2006a). This suggests that the impact of the presence or the absence of a double bond on olfactory detectability may be chemical class-specific.

Finally, we found that the length of the carbon backbone systematically affected olfactory detection thresholds, with both mice and spider monkeys being significantly more sensitive for alcohols with longer-chained (C_8_) compared to shorter-chained (C_4_ or C_5_) alcohols. This finding confirms a general pattern found with aliphatic substances containing an oxygen-containing functional group: in most mammal species tested so far with homologous series of aliphatic substances such as 1-alcohols, *n*-aldehydes, *n*-carboxylic acids, 2-ketones, or *n*-acetic esters, olfactory sensitivity increases with carbon chain length up to C_8_. Such a positive correlation has been reported not only in mice and spider monkeys (Eliasson et al. 2015; Güven and Laska 2012; Hernandez Salazar et al. 2003), but also in other species such as squirrel monkeys (Laska and Seibt 2002, 2003), pigtail macaques (Laska et al. 2003b, 2005b), and human subjects (van Gemert 2011). It is important to note that this correlation between carbon chain length and olfactory sensitivity is not a simple function of vapor pressure as several studies found that aliphatic odorants with carbon backbones longer than C8 are only detected at higher concentrations compared to shorter ones (e.g., Hernandez Salazar et al. 2003; Eliasson et al. 2015). In summary, our findings are at least partially consistent with our second prediction that molecular structural features of the odorants under investigation have a systematic effect on olfactory detectability.

### Behavioral relevance of mold-associated odorants

An increasing number of studies suggest that the behavioral relevance of a given odor stimulus may determine an animal’s olfactory sensitivity for this stimulus. Rats, for example, have been found to be much more sensitive for trimethylthiazoline, a component of the feces of its natural predator, the fox, compared to mammals that are not prey species (Laska et al. 2005a). Similarly, highly frugivorous species such as spider monkeys have been found to detect the main components of fruit odors at markedly lower concentrations as compared to granivorous species such as rats or carnivorous species such as dogs (Hernandez Salazar et al. 2003). Within-species comparisons of olfactory detection thresholds support the notion of a possible link between olfactory sensitivity and behavioral relevance: dogs, for example, are clearly more sensitive for short-chained carboxylic acids than for aliphatic esters (Laska 2017). This should not be surprising considering that the former are the main constituents of the body odor of their prey and thus presumably relevant for a carnivorous species, whereas the latter are plant-derived compounds and thus presumably not, or at least less relevant for dogs.

Among the 85 odorants for which olfactory detection thresholds have been published so far in mice (Laska 2017), there are only eight for which this species is more sensitive than for 1-octen-3-one and 1-octen-3-ol, the mold-associated odorants for which the mice of the present study displayed the lowest threshold values (see Table 1). This suggests that at least these two mold-associated odorants may be behaviorally relevant for mice. A corresponding within-species comparison shows that among the 89 odorants tested so far with spider monkeys (Laska and Hernandez Salazar 2015), the mold-associated odorant with the lowest threshold value in the present study, 3-octanone, ranks only as number 27 concerning its threshold value. This suggests that the mold-associated odorants tested here might not be particularly relevant for the spider monkeys.

However, two caveats should be considered with regard to the supposed link between olfactory sensitivity and behavioral relevance of odor stimuli: first, the eight odorants tested here, although quantitatively predominant in the headspace above various species of mold (Börjesson et al. 1992; Schnürer et al. 1999; Li et al. 2016), are not necessarily those compounds that may trigger avoidance responses during food selection. Several studies have shown that trace components rather than quantitatively predominant components of complex odor mixtures may be responsible for eliciting behavioral responses (e.g., pheromones that are part of body odors, Wyatt 2014). Second, we cannot exclude the possibility that the high sensitivity of the mice for 1-octen-3-one and 1-octen-3-ol might be due to a behavioral context other than the avoidance of mold-infested food. Both compounds have also been reported to naturally occur outside of the biochemical pathways involved in putrefaction, e.g., in certain fruit and vegetable flavors when these are ripe and not infested with mold (Hui 2010). In summary, the present findings partially support our third prediction that olfactory sensitivity is positively linked with the behavioral relevance of the odor stimuli.

A recent study (Peris et al. 2017) reported that fungal infestation increased rather than decreased consumption of oranges (*Citrus sinensis*) by frugivorous non-primate mammals and birds. However, the authors used ripe fruits that were artificially inoculated with *Penicillium digitatum*, and not overripe ones. Thus, it is possible that the honest odor signal of ripeness provided by the fruit itself may have overridden the honest odor signal of putrefaction provided by the mold. Furthermore, the authors did not directly observe consumption of such mold-inoculated fruits by frugivores, but concluded fruit consumption only indirectly from foot tracks and displacement of fruits. This illustrates the complexity of the decision that frugivorous or granivorous species have to make when assessing the palatability of mold-infested food. In any case, it should be emphasized that the vast majority of studies on behavioral responses to mold-infested food report avoidance rather than attraction responses across a wide variety of taxa (Hughes 1990; Pitt and Hocking 2009).

Quantitative studies on the concentrations of mold-associated odorants in the headspace above mold-infested foods are sparse (Börjesson et al. 1992; Schnürer et al. 1999). However, the few quantitative data that are available and the olfactory detection threshold values of the present study suggest that both mice and spider monkeys are clearly able to detect the presence of molds and thus to assess the palatability of potential food using the volatiles produced by molds during putrefaction.

In conclusion, the results of the present study provide useful information for the choice of adequate stimulus concentrations in electrophysiological or imaging studies of the olfactory system or investigations of the discriminative abilities of mice and spider monkeys. Furthermore, they can be used as a basis for establishing dose–response curves in behavioral studies of the attractive or aversive properties of mold-associated odorants. Finally, they contribute to the still rather limited set of data on olfactory sensitivity in nonhuman species which can be used to further test hypotheses concerning the mechanisms underlying between-species differences in olfactory capabilities and the selective pressures acting on olfactory systems.

## References

[CR1] Barkai-Golan R, Paster N (2008). Mycotoxins in fruits and vegetables.

[CR2] Bodyak N, Slotnick B (1999). Performance of mice in an automated olfactometer: odor detection, discrimination and odor memory. Chem Senses.

[CR3] Börjesson T, Stöllman U, Schnürer J (1992). Volatile metabolites produced by six fungal species compared with other indicators of fungal growth on cereal grains. Appl Environ Microbiol.

[CR4] Chambers E, Smith EC, Seitz LM, Sauer DB (1998). Sensory properties of musty compounds in food. Dev Food Sci.

[CR5] Crusio WE, Sluyter F, Gerlai RT, Pietropaolo S (2013). Behavioral genetics of the mouse. Genetics of behavioral phenotypes.

[CR6] Dhanasekaran D, Shanmugapriya S, Thajuddin N, Panneerselvam A, Guevara-Gonzalez RG (2011). Aflatoxins and aflatoxicosis in human and animals. Aflatoxins—biochemistry and molecular biology.

[CR7] Doty RL, Laing DG, Doty RL (2015). Psychophysical measurement of human olfactory function. Handbook of olfaction and gustation.

[CR8] Dykyi J, Svoboda J, Wilhiot RC, Frenkel M, Hall KR (2001). Landolt-Börnstein. Numerical data and functional relationships in science and technology, Group IV.

[CR9] Eliasson M, Hernandez Salazar LT, Laska M (2015). Spider monkeys (*Ateles geoffroyi*) are less sensitive to the odor of aliphatic ketones than to the odor of other classes of aliphatic compounds. Neurosci Res.

[CR10] Forbes SL, Perrault KA (2014). Decomposition odour profiling in the air and soil surrounding vertebrate carrion. PLoS One.

[CR11] Gilad Y, Wiebe V, Przeworski E, Lancet D (2004). Loss of olfactory receptor genes coincides with the acquisition of full trichromatic vision in primates. PLoS Biol.

[CR12] Goff SA, Klee HJ (2006). Plant volatile compounds: sensory cues for health and nutritional value?. Science.

[CR13] Güven SC, Laska M (2012). Olfactory sensitivity and odor structure-activity relationships for aliphatic carboxylic acids in CD-1 mice. PLoS One.

[CR14] Hastings L, Doty RL (2003). Psychophysical evaluation of olfaction in nonhuman mammals. Handbook of olfaction and gustation.

[CR15] Hernandez Salazar LT, Laska M, Rodriguez Luna E (2003). Olfactory sensitivity for aliphatic esters in spider monkeys (*Ateles geoffroyi*). Behav Neurosci.

[CR16] Hughes RN (1990). Behavioural mechanisms of food selection.

[CR17] Hughes GM, Boston ESM, Finarelli JA, Murphy WJ, Higgins DG, Teeling EC (2018). The birth and death of olfactory receptor gene families in mammalian niche adaptation. Mol Biol Evol.

[CR18] Hui YH (2010). Handbook of fruit and vegetable flavors.

[CR19] Janzen DH (1977). Why fruits rot, seeds mold, and meat spoils. Am Nat.

[CR20] Jelen H, Wasowicz E (1998). Volatile fungal metabolites and their relation to the spoilage of agricultural commodities. Food Rev Int.

[CR21] Joshi D, Völkl M, Shepherd GM, Laska M (2006). Olfactory sensitivity for enantiomers and their racemic mixtures—a comparative study in CD-1 mice and spider monkeys. Chem Senses.

[CR22] Kaminski E, Stawicki S, Wasowicz E (1974). Volatile flavor compounds produced by molds of *Aspergillus, Penicillium*, and *Fungi imperfecti*. Appl Microbiol.

[CR23] Keverne EB (2004). Brain evolution, chemosensory processing, and behavior. Nutr Rev.

[CR24] Kjeldmand L, Hernandez Salazar LT, Laska M (2011). Olfactory sensitivity for sperm-attractant aromatic aldehydes: a comparative study in human subjects and spider monkeys. J Comp Physiol A.

[CR25] Kovacevic N, Henderson JT, Chan E, Lifshitz N, Bishop J, Evans AC, Henkelmann RM, Chen XJ (2005). A three-dimensional MRI atlas of the mouse brain with estimates of the average and variability. Cereb Cortex.

[CR26] Larsson L, Laska M (2011). Ultra-high olfactory sensitivity for the human sperm-attractant aromatic aldehyde bourgeonal in CD-1 mice. Neurosci Res.

[CR27] Laska M (2014). Olfactory sensitivity and odor structure-activity relationships for aliphatic ketones in CD-1 mice. Chem Senses.

[CR28] Laska M, Buettner A (2017). Human and animal olfactory capabilities compared. Springer Handbook of Odor.

[CR29] Laska M, Hernandez Salazar LT, Doty RL (2015). Olfaction in nonhuman primates. Handbook of olfaction and gustation.

[CR30] Laska M, Seibt A (2002). Olfactory sensitivity for aliphatic esters in squirrel monkeys and pigtail macaques. Behav Brain Res.

[CR31] Laska M, Seibt A (2003). Olfactory sensitivity for aliphatic alcohols in squirrel monkeys and pigtail macaques. J Exp Biol.

[CR32] Laska M, Hernandez Salazar LT, Rodriguez Luna E (2003). Successful acquisition of an olfactory discrimination paradigm by spider monkeys *(Ateles geoffroyi)*. Physiol Behav.

[CR33] Laska M, Hofmann M, Simon Y (2003). Olfactory sensitivity for aliphatic aldehydes in squirrel monkeys and pigtail macaques. J Comp Physiol A.

[CR34] Laska M, Wieser A, Rivas Bautista RM, Hernandez Salazar LT (2004). Olfactory sensitvitiy for carboxylic acids in spider monkeys and pigtail macaques. Chem Senses.

[CR35] Laska M, Fendt M, Wieser A, Endres T, Hernandez Salazar LT, Apfelbach R (2005). Detecting danger—or just another odorant? Olfactory sensitivity for the fox odor component 2,4,5-trimethylthiazoline in four species of mammals. Physiol Behav.

[CR36] Laska M, Miethe V, Rieck C, Weindl K (2005). Olfactory sensitivity for aliphatic ketones in squirrel monkeys and pigtail macaques. Exp Brain Res.

[CR37] Laska M, Höfelmann D, Huber D, Schumacher M (2006). Does the frequency of occurrence of odorants in the chemical environment determine olfactory sensitivity? A study with acyclic monoterpene alcohols in three species of nonhuman primates. J Chem Ecol.

[CR38] Laska M, Joshi D, Shepherd GM (2006). Olfactory sensitivity for aliphatic aldehydes in CD-1 mice. Behav Brain Res.

[CR39] Laska M, Rivas Bautista RM, Hernandez Salazar LT (2006). Olfactory sensitivity for aliphatic alcohols and aldehydes in spider monkeys *Ateles geoffroyi*. Am J Phys Anthropol.

[CR40] Laska M, Freist P, Krause S (2007). Which senses play a role in nonhuman primate food selection? A comparison between squirrel monkeys and spider monkeys. Am J Primatol.

[CR41] Laska M, Persson O, Hernandez Salazar LT (2009). Olfactory sensitivity for alkylpyrazines – a comparative study in CD-1 mice and spider monkeys. J Exp Zool A.

[CR42] Li N, Alfiky A, Vaughan MM, Kang S (2016). Stop and smell the fungi: fungal volatile metabolites are overlooked signals involved in fungal interaction with plants. Fungal Biol Rev.

[CR43] Løtvedt PK, Murali SK, Hernandez Salazar LT, Laska M (2012). Olfactory sensitivity for “green odors” (aliphatic C6 alcohols and C6 aldehydes)—a comparative study in male CD-1 mice *(Mus musculus)* and female spider monkeys *(Ateles geoffroyi)*. Pharmacol Biochem Behav.

[CR44] Morris CF, McLean D, Engleson JA, Fuerst EP, Burgos F, Coburn E (2012). Some observations on the granivorous feeding behavior preferences of the house mouse (*Mus musculus* L.). Mammalia.

[CR45] Murphy PA, Hendrich S, Landgren C, Bryant CM (2006). Food mycotoxins: an update. J Food Sci.

[CR46] Nei M, Niimura Y, Nozawa M (2008). The evolution of animal chemosensory receptor gene repertoires: roles of chance and necessity. Nat Rev Genet.

[CR47] Nevo O, Valenta K (2018). The ecology and evolution of fruit odor: implications for primate seed dispersal. Int J Primatol.

[CR48] Nevo O, Orts Garri R, Hernandez Salazar LT, Schulz S, Heymann EW, Ayasse M, Laska M (2015). Chemical recognition of fruit ripeness in spider monkeys *(Ateles geoffroyi)*. Sci Rep.

[CR49] Nummela S, Pihlström H, Puolamäki K, Fortelius M, Hemilä S, Reuter T (2013). Exploring the mammalian sensory space: co-operations and trade-offs among senses. J Comp Physiol A.

[CR50] Pablo-Rodriguez M, Hernandez-Salazar LT, Aureli F, Schaffner CM (2015). The role of sucrose and sensory systems in fruit selection and consumption of *Ateles geoffroyi* in Yucatan, Mexico. J Trop Ecol.

[CR51] Pearce JM (2008). Animal learning and cognition.

[CR52] Peris JE, Rodriguez A, Peña L, Fedriani JM (2017). Fungal infestation boosts fruit aroma and fruit removal by mammals and birds. Sci Rep.

[CR53] Pitt JI, Hocking AD (2009). Fungi and food spoilage.

[CR54] Pomeroy SL, La Mantia AS, Purves D (1990). Postnatal construction of neural circuitry in the mouse olfactory bulb. J Neurosci.

[CR55] Rouquier S, Giorgi D (2007). Olfactory receptor gene repertoires in mammals. Mutat Res.

[CR57] Sarrafchi A, Odhammer AME, Hernandez Salazar LT, Laska M (2013). Olfactory sensitivity for six predator odorants in CD-1 mice, human subjects, and spider monkeys. PLoS One.

[CR58] Schnürer J, Olsson J, Börjesson T (1999). Fungal volatiles as indicators of food and feeds spoilage. Fungal Genet Biol.

[CR59] Shepherd GM (2005). Outline of a theory of olfactory processing and its relevance to humans. Chem Senses.

[CR60] Stephan H, Baron G, Frahm HD, Steklis HD, Erwin J (1988). Comparative size of brains and brain components. Comparative primate biology.

[CR61] Stephens DW, Krebs JR (1986). Foraging theory.

[CR62] Stoddart DM, Stoddart DM (1980). Detection of food. The ecology of vertebrate olfaction.

[CR63] van Gemert LJ (2011). Odour thresholds. Compilations of odour threshold values in air, water and other media.

[CR64] Vass AA, Smith RR, Thompson CV, Burnett MN, Wolf DA, Synstelien JA, Dulgerian N (2004). Decomposition odor analysis database. J Forensic Sci.

[CR65] Wallén H, Engström I, Hernandez Salazar LT, Laska M (2012). Olfactory sensitivity for six amino acids: a comparative study in CD-1 mice and spider monkeys. Amino Acids.

[CR66] Weast RC (1987). Handbook of chemistry and physics.

[CR67] Wong ZA, Hsieh DPH (1980). The comparative metabolism and toxicokinetics of aflatoxin B_1_ in the monkey, rat, and mouse. Toxicol Appl Pharmacol.

[CR68] Wyatt TD (2014). Pheromones and animal behavior.

